# Anaesthetic and Procedural-Related Complications in Transcatheter Aortic Valve Implantation (TAVI) and Its Outcome: A Retrospective Observational Study

**DOI:** 10.7759/cureus.70975

**Published:** 2024-10-07

**Authors:** Akbar Mistry, Muhammad S Yousuf, Khalid M Siddiqui, Osman Fahim, Saulat Fatimi, Khalid Samad

**Affiliations:** 1 Anaesthesiology, Aga Khan University Hospital, Karachi, PAK; 2 Cardiology, Aga Khan University Hospital, Karachi, PAK; 3 Cardiothoracic Surgery, Aga Khan University Hospital, Karachi, PAK; 4 Anesthesia and Critical Care, Aga Khan Health Service, Pakistan, Karachi, PAK

**Keywords:** anaesthesia, aortic stenosis, procedure-related complications, severe aortic stenosis, transcatheter aortic valve implantation

## Abstract

Background

Transcatheter aortic valve implantation (TAVI) presents significant challenges in its management, not only due to the technical complexity of the procedure but also because it is primarily performed on elderly patients who often have multiple comorbidities, making perioperative care and post-procedural recovery more intricate and demanding. The study's objective is to discover the challenges faced during the TAVI procedure and the frequency of complications that occurred during and after the procedure.

Methods

This is a single-centre retrospective study. Patients with symptomatic severe aortic stenosis, considered at high risk for conventional surgical aortic valve replacement, were included, their medical records were extracted, and data were collected. Endpoints at one-year follow-up comprised one-year mortality, cause of death, and valve dysfunction. Procedural-related complications were also noted as procedural-related outcomes.

Results

The mean age and body mass index (BMI) of the patients were 73.9 years and 28.3 kg/m^2^, respectively. The main comorbidities were hypertension (n = 33, 84.6%), diabetes mellites (n = 21, 53.8%), ischemic heart disease (n = 6, 15.38%), and chronic obstructive pulmonary disease (COPD) (n = 5, 12.8%). The mean European System for Cardiac Operative Risk Evaluation (EuroSCORE) was 5.75% with a median of 2.38%. General anaesthesia was received (n = 27), whereas the rest of the cases were performed under monitored anaesthesia care (MAC). None of the patients developed surgical site hematoma or massive blood loss. Post-operative mechanical ventilation was required in three patients (10.2%), and two (5.12%) of them expired in the intensive care unit. One patient had on-table mortality.

Conclusion

A low rate of anaesthesia and procedural-related complications was observed. The TAVI procedures done in our centre are promising and parallel to the finest global centres' outcomes in terms of procedural triumph and complication rate.

## Introduction

The incidence of aortic valvular diseases is on the rise all around the world. The incidence has increased with people living a prolonged life [1]. The leading cause of aortic valvular diseases is rheumatic fever, but infective endocarditis due to other causes and congenital bicuspid aortic valves cause a vast number of aortic lesions. The burden of rheumatic valvular diseases is disproportionate, being more in the underdeveloped countries as compared to the developed ones. With increasing age, the incidence of aortic stenosis increases [2].

Conventionally, aortic valve replacements have been performed via sternotomy. Options for valve replacements include bioprosthetic and mechanical, with mechanical being longer lasting. However, with increasing age and the presence of aortic disease, patients come with a variety of comorbidities, and therefore an open approach itself poses a risk to the patient. In 2002, the first-ever transcatheter aortic valve replacement was performed using a percutaneous heart valve. It was observed that this approach was more suited for patients with multiple other comorbidities like hypertension, coronary artery disease (CAD), heart failure, chronic kidney disease (CKD), and diabetes mellitus with a shorter life expectancy. The risk of sepsis, infection, prolonged mechanical ventilation, acute kidney injury, and on-table death has been lower in patients undergoing transcatheter aortic valve implantation (TAVI) compared to those who undergo open valve replacements. Although anaesthesia-related complications have been significantly reduced in the last few years. In the instance any challenges from the procedure arise, it could cause an administrative and financial challenge due to the possibility of extended hospital stays and increased healthcare expenses [3-6].

The main purpose of this study was to discover the challenges faced during the procedure and the frequency of complications that occurred during and after the procedure. The secondary objective is to find out the clinical outcomes of TAVI.

## Materials and methods

This study is a single-centre retrospective analysis, focusing on patients who underwent TAVI, in Aga Khan University Hospital**, **Karachi, Pakistan. The data for the study were sourced from TAVI procedures conducted over a four-year period, from November 2017 to November 2021. Following the exemption from ethical approval by the institution's Ethics Review Committee, relevant medical records were systematically identified, extracted, and thoroughly reviewed. 

The study population consisted of patients diagnosed with symptomatic severe aortic stenosis, who were considered at high risk for conventional surgical aortic valve replacement due to various clinical or anatomical factors. These high-risk patients were included based on established clinical criteria for severe aortic stenosis, such as reduced aortic valve area and elevated transvalvular pressure gradients. To maintain data integrity and ensure reliable outcomes, patients with incomplete or missing medical records pertinent to the TAVI procedure were excluded from the study.

Procedural-related complications like myocardial infarction, major bleeding, cerebrovascular accidents, difficulty in vascular access and acute kidney injury were also noted during the procedure.

Statistical analysis

After data were collected, they were entered into IBM SPSS Statistics for Windows, Version 29.0.2.0 (released 2023, IBM Corp., Armonk, NY). The analysis included the distribution of the patients in terms of age, gender, and BMI. The median EUROSCORE was compared with respect to post-operative complications and morbidity. Modalities used to administer anaesthesia and monitoring techniques were plotted against complications. The incidence of various complications such as airway-related, procedure-related, and cardiac events was calculated. Subgroup analysis of the incidence of complications with respect to age, gender and other co-morbidities was also performed.

## Results

A total of 39 cases were performed between the years 2017 and 2021. There was a male preponderance according to the analysis, with 27 males and 12 females. The mean age of the patients was 73.9 ± 9.59 years while the mean BMI was 28.3 kg/m^2^. The distribution of patients and procedural characteristics are shown in Table 1. One of the patients had undergone a redo TAVI. The mean EURO score was 5.75% with a median of 2.38%. All the patients were classified as the American Society of Anesthesiologists IV (ASA IV). An Apfel score of 2 was found in 20% of the patients while the rest had 0 or 1, but postoperative nausea was found in only one of the patients.

**Table 1:** Baseline characteristics of patients (n = 39) Apfel Score (postoperative nausea and vomiting (PONV) risk assessment tool) score from 0 to 4 (0 = no PONV risk, 1 = low PONV risk, 2 = moderate PONV risk and 3-4 = high PONV risk)

Demographic variables	Values
Age (years) mean ± SD	73.9 ± 9.59
Gender	
Female, n (%)	12 (30.8%)
Male, n (%)	27 (69.2%)
Body mass index (BMI) median [Q1, Q3]	28.5 [24.0, 30.6]
Comorbidities	
Hypertension, n (%)	33 (84.6%)
Diabetes mellitus, n (%)	21 (53.8%)
Ischemic heart disease (IHD), n (%)	36 (92.2%)
Chronic obstructive pulmonary disease (COPD), n (%)	5 (12.8%)
Mode of anaesthesia	
General anaesthesia (GA), n (%)	28 (71.8%)
Monitored anesthesia care (MAC), n (%)	11 (28.2%)
Baseline investigations	
Hemoglobin (g/dL) mean ± SD	11.2 ± 1.8
Aortic valve area (cm²) mean ± SD	0.69 ± 0.17
Non-invasive gradient (mmHg), mean ± SD	44.1 ± 15.2
Ejection fraction (%) mean ± SD	51.6 ± 12.7
Risk assessment of procedural morbidity and mortality	
EUROSCORE II median [Q1, Q3]	2.38 [1.61, 5.29]
Apfel score *	
0, n (%)	7 (17.9%)
1, n (%)	21 (53.8%)
2, n (%)	8 (20.5%)
3-4, n (%)	3 (7.7%)
Procedural characteristics	
Access site	
Femoral, n (%)	39 (100%)
Device	
CoreValve, n (%)	37 (94.8%)
Edward Sapien, n (%)	2 (5.1%)
Invasive mean gradient	
Pre-TAVI (mmHg) mean ± SD	43.7 ± 19.1
Post-TAVI (mmHg) mean ± SD	1.9 ± 3.3
Aortic regurgitation index mean ± SD	28.0 ± 7.8

General anaesthesia was opted for 70% of the patients, whereas the rest of the cases were performed under MAC. Etomidate was used for induction in five out of 39 patients, whereas propofol was used for the rest. A pre-induction arterial line was taken for all the patients, whereas central line insertion was done for 12 out of 39 patients (30.76%). Intraoperative hypotension occurred in 67.6% of the patients, whereas hypothermia was found in 12.8%.

None of the patients developed surgical site hematoma or massive blood loss. Only one procedure was switched to an open heart. 10.8% of the patients were kept on the ventilator post-operatively, whereas two of them expired in the intensive care unit. Clinical outcomes are listed in Table 2.

**Table 2:** Clinical outcomes of transcatheter aortic valve implantation (TAVI)

Clinical outcomes	n (%)
Any cause mortality	1 (2.5%)
Cardiovascular mortality	1 (2.5%)
Valve dysfunction	0
Pacemaker implantation	0
Bleeding	
Major > 500 ml	1 (2.5%)
Minor <500 ml	3 (7.6%)
Vascular complications	
Major	0
Minor	2 (5.1%)
Hypothermia	5 (12.8%)
Allergic reaction	1 (2.5%)
Acute kidney injury (< 72 h)	
Stage 1	3 (7.6%)
Stage 2	0
Stage 3	0
Myocardial infarction (<72 hours)	1 (2.5%)
Cerebrovascular accident	1 (2.5%)
On table death	1 (2.5%)

## Discussion

Aortic valve stenosis is a debilitating condition in the elderly, which often necessitates open surgical replacement. If left untreated, it causes progressive worsening and symptoms such as shortness of breath, syncope, and chest pain [7]. Being a high-risk procedure for patients with advanced age and multiple comorbidities, open surgical valve replacement carries a high risk, and that is why TAVI is now being offered to such patients [8]. Criber et al. performed the first ever TAVR, after which there has been a significant improvement in quality of life for patients with severe aortic diseases [9]. The technique has since then been modified, and simplified and newer valves have been introduced to make this procedure safer for high-risk patients [10].

Two trials were performed to assess the feasibility and rate of complications in the initial phases, namely, I-REVIVE and RECAST [11,12]. These trials included a total of 35 patients with a success rate of 75% and 30-day mortality of 23.1%. On the other hand, the German registry showed a success rate of 98.4% with a 30-day mortality of 12.4% [13]. In our analysis, the success rate of TAVR is reported to be 95%. Thirty-day mortality however could not be calculated as many patients lost follow-up. One of the patients died on the table, while one died in the intensive care unit (ICU).

A meta-analysis was performed that included four randomized controlled trials comparing patients undergoing open surgical aortic valve replacement versus trans aortic valve replacement (TAVR), which showed a significant decrease in post-procedure complications, including lower mortality and risk of bleeding [14]. Another systematic review is based on 11 different studies comparing open surgical aortic valve replacement to TAVR. This review concluded that most of the studies showed equivocal results in terms of post-procedure mortality between open surgical repair versus TAVR. Significant differences stated that the incidence of acute renal injury, complications of bleeding and atrial fibrillation were less seen with TAVR, whereas those undergoing open surgical replacement had lower incidence of pacemaker dependency and paravalvular regurgitation [15]. Our review showed that post-procedure hematoma developed in only one out of 39 patients.

A meta-analysis published in 2014 compared complications in patients undergoing TAVR under general anaesthesia versus those undergoing the procedure under MAC with local anaesthesia infiltration at the cannulation site. This included a total of 1,524 patients. The reviews showed that patients undergoing TAVR under MAC had less hospital stay, but there was no significant difference in short-term outcomes such as acute renal failure and surgical site hematoma [16].

Intraoperative hypothermia is another factor that leads to complications such as coagulopathy, rhythm abnormalities, and delayed recovery. Our study showed the incidence of hypothermia to be 12.5% owing to the lack of active warming devices in the cath lab at our centre. A study compared the use of forced air warming devices versus a water circulating device, showing that the water circulating technique was superior to the standard forced air warming method, with the lowest reported temperature to be 36.01 ± 0.58 versus 34.89 ± 0.76°C, p < 0.001 [17]. Our study showed that the lowest reported temperature for patients undergoing TAVR under general anaesthesia was 34.2°C, and for those undergoing the procedure under MAC, the temperature was either not recorded or a skin probe was used.

Postoperative nausea and vomiting were absent in all the patients. However, four out of 39 patients required mechanical ventilation for more than 24 hours in the ICU, which also suggests that MAC with local infiltration at the cannulation site could serve as a better option for high-risk patients. Two of the patients developed allergic reactions to either the contrast injected, or the antibiotics, but this did not lead to adverse reactions such as anaphylaxis. Airway-related trauma was virtually absent in all the procedures, which could be due to the presence of senior anaesthesia trainees and consultants, with video laryngoscope readily available for all of the cases.

The study's strength is that all patients received treatment for TAVI following a multidisciplinary team review carried out by our institution's cardiac anaesthetists, cardiologists, and cardiac surgeons. This study's description of the whole learning curve and early experience is another noteworthy feature.

This study has a few limitations. As a single-centre retrospective study, the findings may not be generalizable to other populations, limiting external validity. The small sample size may reduce the ability to detect less common complications. Lastly, the one-year follow-up period may not capture long-term outcomes or complications.

It is felt that sharing local experiences and outcomes would be crucial to provide a roadmap to centres planning to start the TAVI program speciality where the resources are limited. Based on the results of the single centre, we would recommend that the treatment strategy that works very well in one centre may not necessarily be the same in another.

Limitations

This study has a few limitations. As a single-centre retrospective study, the findings may not be generalizable to other populations, limiting external validity. The small sample size may reduce the ability to detect less common complications. Lastly, the one-year follow-up period may not capture long-term outcomes or complications.

## Conclusions

The TAVI procedures performed at our centre demonstrate outcomes that are both promising and comparable to those reported by leading global institutions, particularly in terms of procedural success and complication rates. However, a multi-centre study with a longer follow-up, such as one year, would provide a more comprehensive evaluation of complications and enable the development of optimized strategies for cardiac anesthesiologists. Such research is crucial for advancing patient care in high-risk populations. These findings may encourage further exploration of the evolving role of TAVI in managing aortic stenosis and inspire continued innovation in perioperative care.
